# Using Sweet Sorghum Varieties for the Phytoremediation of Petroleum-Contaminated Salinized Soil: A Preliminary Study Based on Pot Experiments

**DOI:** 10.3390/toxics11030208

**Published:** 2023-02-23

**Authors:** Di Ma, Jie Xu, Jipeng Zhou, Lili Ren, Jian Li, Zaiwang Zhang, Jiangbao Xia, Huicheng Xie, Tao Wu

**Affiliations:** 1 Shandong Key Laboratory of Eco-Environmental Science for the Yellow River Delta, Shandong Provincial Engineering and Technology Research Center for Wild Plant Resources Development and Application of Yellow River Delta, College of Biological and Environmental Engineering, Binzhou University, Binzhou 256603, China; 2 College of Forestry, Shandong Agricultural University, Taian 271018, China; 3 Department of Bioengineering, Binzhou Vocational College, Binzhou 256600, China

**Keywords:** *Sorghum bicolor*, petroleum hydrocarbon, removal, energy plants

## Abstract

Using energy plants to repair salinized soils polluted by petroleum is an efficient way to solve the problem of farmland reduction and prevent pollutants from entering the food chain simultaneously. In this study, pot experiments were conducted for the purposes of preliminarily discussing the potential of using an energy plant, sweet sorghum (*Sorghum bicolor* (L.) Moench), to repair petroleum-polluted salinized soils and obtain associated varieties with excellent remediation performance. The emergence rate, plant height and biomass of different varieties were measured to explore the performance of plants under petroleum pollution, and the removal of petroleum hydrocarbons in soil with candidate varieties was also studied. The results showed that the emergence rate of 24 of the 28 varieties were not reduced by the addition of 1.0 × 10^4^ mg/kg petroleum in soils with a salinity of 0.31%. After a 40-day treatment in salinized soil with petroleum additions of 1.0 × 10^4^ mg/kg, 4 potential well-performed varieties including Zhong Ketian No. 438, Ke Tian No. 24, Ke Tian No. 21 (KT21) and Ke Tian No. 6 with a plant height of >40 cm and dry weight of >4 g were screened. Obvious removal of petroleum hydrocarbons in the salinized soils planted with the four varieties were observed. Compared with the treatment without plants, the residual petroleum hydrocarbon concentrations in soils planted with KT21 decreased by 69.3%, 46.3%, 56.5%, 50.9% and 41.4%, for the additions of 0, 0.5 × 10^4^, 1.0 × 10^4^, 1.5 × 10^4^ and 2.0 × 10^4^ mg/kg, respectively. In general, KT21 had the best performance and application potential to remediate petroleum-polluted salinized soil.

## 1. Introduction

In recent years, soil petroleum pollution has become one of the most important environmental problems to be solved, with a background of farmland reduction and energy shortages on a global scale [1]. In some regions with salinized soils, petroleum production bases exist and various chemicals enter the soil during the process of oil exploitation, resulting in petroleum pollution of the salinized soil [2,3,4]. In salinized soils, the solubility of petroleum hydrocarbons in the soil water is low, leading to the slow dispersion of these pollutants in the soil, reducing carbon sources for common microorganisms, resulting in a lower biological availability of hydrocarbons in high-salt environments [5]. Petroleum pollution deteriorates the structure of salinized soil, causing nutrient deficiency and thus affecting the chemical and biological processes in the soil [6]. Commonly, physical, chemical and biological methods have been used to remediate petroleum-contaminated soils [7]. Among them, phytoremediation technology, which uses plants and associated microorganisms to remove pollutants from the environmental matrix, is of more concern due to its advantages such as low cost, environmental friendliness, sustainable utilization and so on [1,2]. Therefore, it has become a frontier research field for environmental remediation technology and engineering [8].

Currently, the application of energy plants in soil-contamination remediation has attracted more and more public attention [9]. It conforms to a principle of not competing with people for food or with grain for land and can benefit from remediation at the same time, opening up a new way for the treatment and rational use of contaminated soil [10]. Sweet sorghum (*Sorghum bicolor* (L.) Moench), a species belongs to family Poaceae, genus Sorghum, is one of the most important crop species in salinized alkali soil. It has great adaptability and some useful qualities, such as drought resistance, waterlogging resistance, salinized alkali resistance and high temperature resistance [11]. In addition, it is recognized as an important energy plant for ethanol production, based on sugar and starch fermentation, as well as cellulose fermentation [12,13,14]. Previous studies have reported using *S. bicolor* to repair soils polluted by heavy metals [15,16,17]. However, little information is available for its potential to repair soils contaminated by petroleum pollutants [18]. Poaceae and leguminous plant species are widely used plants for the remediation of petroleum-contaminated soils [19]. At present, many sweet sorghum varieties are produced for commercial use and some of them might be potential phytoremediation tools for petroleum-soil remediation. Thus, it is essential to discover their resistance to petroleum pollution and their capacity to remove those pollutants from soils. 

The main purposes of this study are to: (1) explore the effects of petroleum on the growth of different varieties of *S. bicolor*; (2) compare the capacities of candidate varieties to remove petroleum hydrocarbons from salinized soils; and (3) obtain superior varieties suitable for the phytoremediation of petroleum-contaminated saline soils.

## 2. Materials and Methods

### 2.1. Test Materials

Twenty-eight saline-tolerant varieties of *S. bicolor* were selected as materials (Table 1). Among them, Jiliang No. 3, Zhong Ketian No. 438, Zhong Ketian No. 968, Jinnuo No. 3, Jitian No. 3, Longza No. 11, Ji Tianza No. 2 and Liaotian No. 6 were provided by Crop Research Institute of Shandong Academy of Agricultural Sciences.Gao Dancao, Cao Gaoliang, JT No. 47, JT No. 62, Ketian No. 2, BJ No. 285, Ketian No. 11, Ketian No. 24, J No. 25, Ketian No. 30, JT No. 110, Ketian No. 21, Ketian No. 9, JT No. 98, Ketian No. 17, 2031, J100, WSC29, Ketian No. 6 and Ketian No. 4 were from the Institute of Botany, Chinese Academy of Sciences. 

### 2.2. Test Soil

The soil used in the test was taken from the surface soil (0~20 cm) in the salinized area of the Yellow River Delta. It was salinized fluvo-aquic soil and its basic physical and chemical properties are presented in Table 2. Soil pH and salinity were determined in a soil–water suspension (1:2.5) using an electrode method. The content of organic carbon in soil was measured using a vario EL III analyzer (Elementar, Hanau, Germany). Total N content in soil samples was also detected using the vario EL III analyzer. Available N was extracted using 2 M KCl and detected using a flow AA3 analyzer (Seal, Norderstedt, Germany). Total P was determined by an Optima 8000 ICP-OES (PerkinElmer, Waltham, MA, USA) after the soil samples were digested using aqua regia and HF [20]. The available P was extracted by 0.5 M NaHCO_3_ and then measured using the molybdenum blue method. The contents of petroleum hydrocarbons (C_10_-C_40_) in soil samples were determined according to the related national standard method of China (HJ1021-2019) using gas chromatography. In short, the soil sample was Soxhlet-extracted with acetone/hexane (*v*/*v* = 1:1) for 18 h. The extract was concentrated using a R-300 rotary evaporator (Buchi, Flawil, Switzerland) and then purified by a magnesium silicate column. The column was eluted with 12 mL hexane. The eluate was concentrated to 1.0 mL and detected for petroleum hydrocarbon using GC-2014 gas chromatography (Shimadzu, Kyoto, Japan). The content of petroleum hydrocarbons in the original soils was 146.67 mg/kg, much lower than the national risk screening value (500 mg/kg) of China.

The petroleum added to the soil was supplied by Gudong Oil Production Plant of Sinopec Shengli Oilfield Company. Its density was 0.89 g/cm^3^ and the mass fraction of petroleum hydrocarbon was 79%.

The preparation method of petroleum-contaminated soil was as follows. After air-dried for 4 days, the soil samples passed through a 2 mm sieve and mixed homogeneously. Petroleum dissolved in dichloromethane was added to a certain amount of soil and fully mixed. In general, concentrations of petroleum in contaminated soil are below 2.0 × 10^4^ mg/kg, at which level phytoremediation can function well. Thus, the addition of petroleum to the prepared soils we set were 0, 5.0 × 10^3^, 1.0 × 10^4^, 1.5 × 10^4^ and 2.0 × 10^4^ mg/kg, respectively.

### 2.3. Screening of S. bicolor Varieties Resistant to Petroleum Pollution

Pot experiments were conducted to explore the effects of petroleum on the growth of different *S. bicolor* varieties. Seeds of different varieties were planted in pots with a diameter of 15 cm and a height of 15 cm. A total of 56 treatments at two addition levels of 0 and 1.0 × 10^4^ mg/kg for petroleum were set and each treatment was repeated three times. In detail, 5 kg of soil was added to the pot, then 30 seeds that had been pre-soaked in water for 10 h were arranged neatly in the pot. After that, the seeds were watered using a rubber-tip dropper, making the bed surface wet; the wet status was kept for 7 days. During this process, rotten seeds were removed once observed. After the 7-day treatment, the number of seedlings in each pot was calculated. After emergence, 3 plants were kept in each pot (removing the others) and cultivated for 40 days. During the growth period, 1000 mg/L of urea aqueous solution prepared with deionized water was used for irrigation, with a frequency of once every 2 weeks and a dosage of 50 mL each time. The pot experiments were conducted in the greenhouse in Binzhou University from March to May. The field capacity was kept at about 60%. After the 40-day growth, the height, fresh weight of the above-ground part and below-ground part of each plant were measured. The dry weight of the plant parts was determined using a gravimetric method.

### 2.4. Phytoremediation Test

Based on the results of the screening experiments, ZK438, KT24, KT21 and KT6, which have strong resistance to petroleum pollution, were selected as the remediation varieties. The tests were also carried out in pots with diameter of 15 cm and height of 15 cm. The five addition levels of petroleum of blank, 5.0 × 10^3^, 1.0 × 10^4^, 1.5 × 10^4^ and 2.0 × 10^4^ mg/kg were set and the soil was added to the pots. A total of 20 treatments were conducted and each one was repeated three times. The methods of soil filling, sowing and emergence calculating were the same as those in Section 2.3. After emergence, three plants were kept in each pot. A 70-day cultivation was then conducted in the greenhouse. These experiments were carried out from August to October, 2022. After finishing the cultivation, the height, fresh weight of the above-ground and below-ground parts and dry weight of the plants were measured as described previously. 

Petroleum hydrocarbon (especially C_10_-C_40_) is the most abundant component of petroleum that can cause a great environmental hazard (Prince et al., 2003). Recommended/standard values of petroleum hydrocarbons (C_10_-C_40_) in soil were also suggested by several countries to evaluate the ecological, environmental risks caused by petroleum pollutants [21,22,23]. In this study, removing an amount of petroleum hydrocarbons (C_10_-C_40_) was used to assess the effects of different remediation varieties. The contents of petroleum hydrocarbons (C_10_-C_40_) in soil samples were determined according to the related national standard method of China (HJ1021-2019) using gas chromatography.

### 2.5. Data Processing

The variance and significance tests of related parameters between treatments were performed using SPSS v20.0 software (SPSS Inc., Chicago, IL, USA). Significant differences were defined as *p* < 0.05.

## 3. Results

### 3.1. Parameters of the Plants in Petroleum-Polluted Salinized Soils

**Figure 1:**
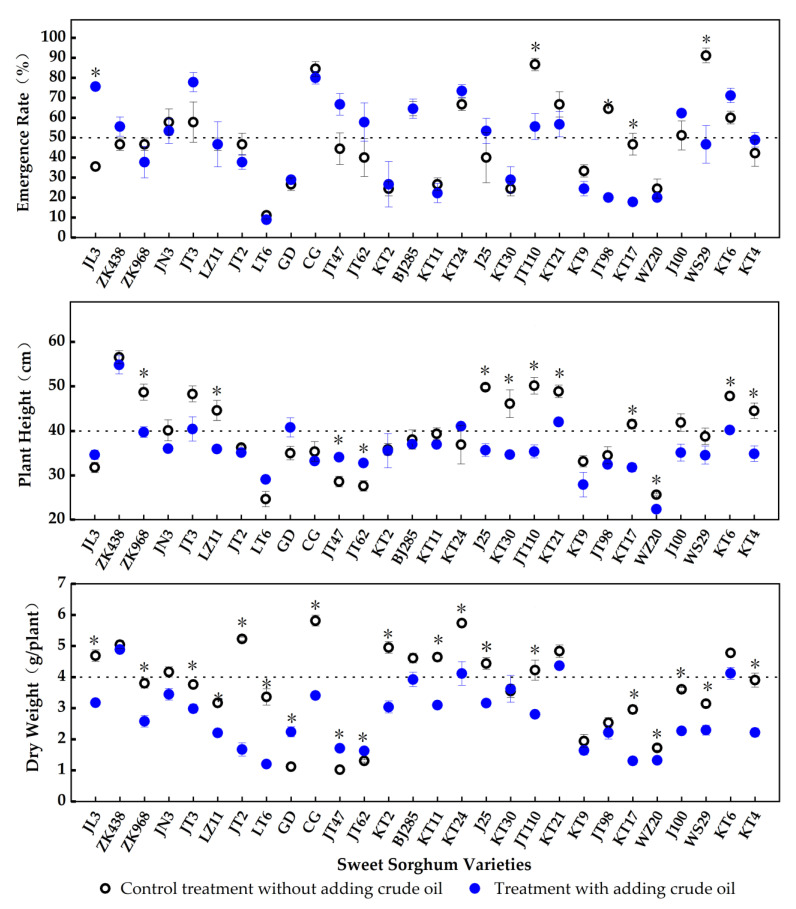
The effect of petroleum-polluted salinized soil (1.0 × 10^4^ mg/kg) on the growth of 28 *S. bicolor* varieties. (*: the variance of a related parameter for a certain *S. bicolor* variety between the petroleum-added treatment and the control group is significant at a *p* < 0.05 level.)

The addition of petroleum had different effects on the emergence rate of different varieties of *S. bicolor* (Figure 1). The addition of petroleum (1.0 × 10^4^ mg/kg) in 0.31% salinized soil significantly reduced the emergence rate of JT110, JT98, KT17 and WS29 as well as increased the emergence rate of JL3 (*p* < 0.05). The variances of emergence rate among the other 23 were not significant (*p* > 0.05). It was worth mentioning that after the 7-day treatment, the emergence rates of *S. bicolor* JL3, ZK438, JN3, JT3, CG, JT47, JT62, BJ285, KT24, J25, JT110, KT21, J100 and KT6 were higher than 50%.

Petroleum pollution had different effects on plant height of different varieties of *S. bicolor* (Figure 1). The plant heights of ZK968, LZ11, J25, KT30, JT110, KT21, KT17, WZ20, KT6 and KT4 in the treatment groups were significantly lower than those of the control groups (*p* < 0.05). Meanwhile, the plant heights of JT47 and JT62 were significantly higher in the treatments than those in the control groups (*p* < 0.05). Among them, ZK438, JT3, GD, KT24, KT21 and KT6 were observed with plant heights greater than 40 cm after being cultured in the petroleum-added salinized soils for 40 days.

The effects of petroleum addition on the biomass of different varieties of *S. bicolor* were different (Figure 1). The dry weights of the plants of JL3, ZK968, JT3, LZ11, JT2, LT6, CG, KT2, KT11, KT24, J25, JT110, KT17, WZ20, J100, WS29 and KT4 in the treatment groups were significantly lower than the associated control groups, and those of CD, JT47 and JT62 in petroleum-added soil were higher than those in soil without petroleum addition (*p* < 0.05). The variances of biomass between the treatment groups and related control groups for ZK438, JN3, BJ258, KT30, KT21, KT9, JT98 and KT6 were insignificant (*p* > 0.05). The above observations suggested CD, JT47, JT62, ZK438, JN3, BJ258, KT30, KT21, KT9, JT98 and KT6 had a certain tolerance to petroleum pollution. In the soil with 1.0 × 10^4^ mg/kg of petroleum added, ZK438, KT24, KT21 and KT6 had a dry weight of >4 g, plant height of >40 cm and emergence rate of >50%, indicating they had a certain tolerance to petroleum pollution in salinized soil. Therefore, ZK438, KT24, KT21 and KT6 were selected for further research.

### 3.2. Growth of the Four Well-Performed Varieties

**Figure 2:**
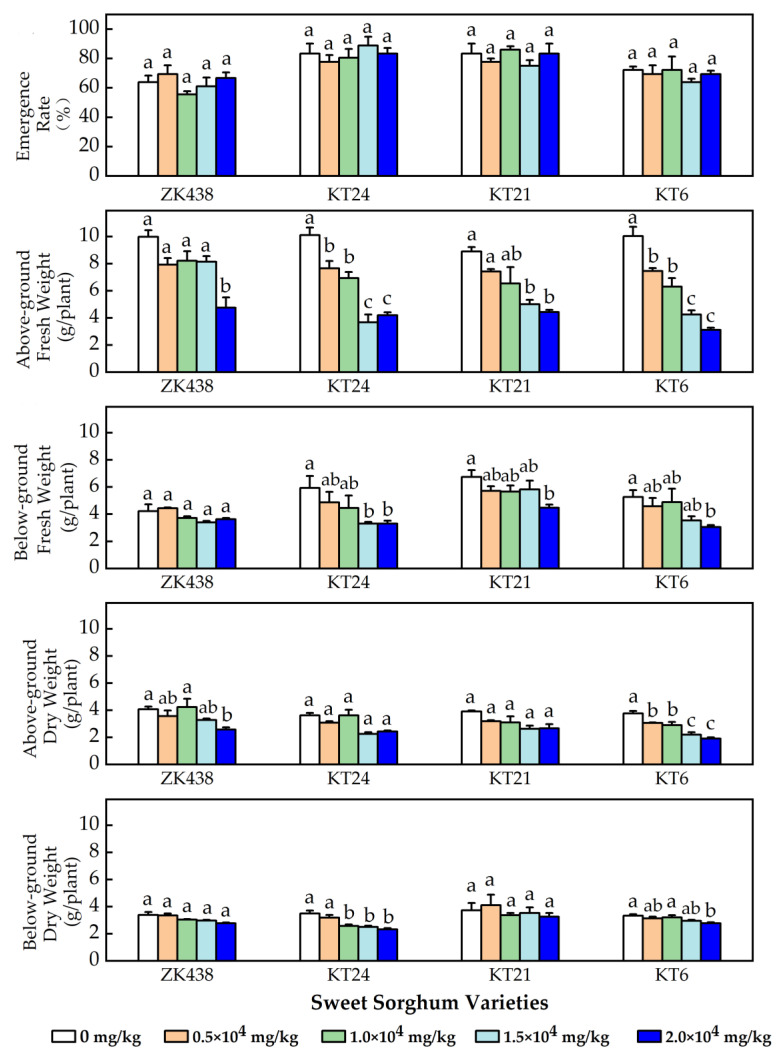
The effects of different contents of petroleum hydrocarbons in salinized soil on the growth of four varieties. (Different letters within the same *S. bicolor* variety indicated significant variances (*p* < 0.05) among the different petroleum addition treatments.)

As shown in Figure 2, the addition of petroleum with contents of 0, 0.5 × 10^4^, 1.0 × 10^4^, 1.5 × 10^4^ and 2.0 × 10^4^ mg/kg in the salinized soil had no significant effect on the emergence rate of the four varieties (*p* > 0.05). The fresh and dry weights of four varieties were differently affected by the contents of crude oils of petroleum in salinized soil (Figure 2). Among the treatment of 0, 0.5 × 10^4^, 1.0 × 10^4^, 1.5 × 10^4^ and 2.0 × 10^4^ mg/kg, the differences in the fresh and dry weight of the underground parts of ZK438 were insignificant (*p* > 0.05), and the differences of the dry weight of both the above-ground parts and the underground parts of KT21 were also not significant (*p* > 0.05). If just considering the additions of petroleum at the levels of 0, 0.5 × 10^4^, 1.0 × 10^4^ and 1.5 × 10^4^ mg/kg, all the weight parameters of ZK438 were without significant differences among the treatments (*p* > 0.05), and the differences of the below-ground fresh weight of KT21 were also insignificant (*p* > 0.05). The above results indicated that ZK438 and KT21 seemed barely affected by the petroleum addition, showing strong tolerance to petroleum in salinized soil. In contrast, KT24 and KT6A seemed to be highly affected by petroleum levels. For example, the fresh weight of the above-ground part of KT24, and the fresh weight of the above-ground part and the dry weight of the underground part of KT6A, were significantly different from their associated control groups at the 0.5 × 10^4^ mg/kg level (*p* < 0.05). KT24 and KT6 had relatively lower tolerance to oil pollution in salinized soils than ZK438 and KT21.

### 3.3. Removal of Petroleum Hydrocarbons in Soils after the Planting of Four S. bicolor Varieties

**Figure 3:**
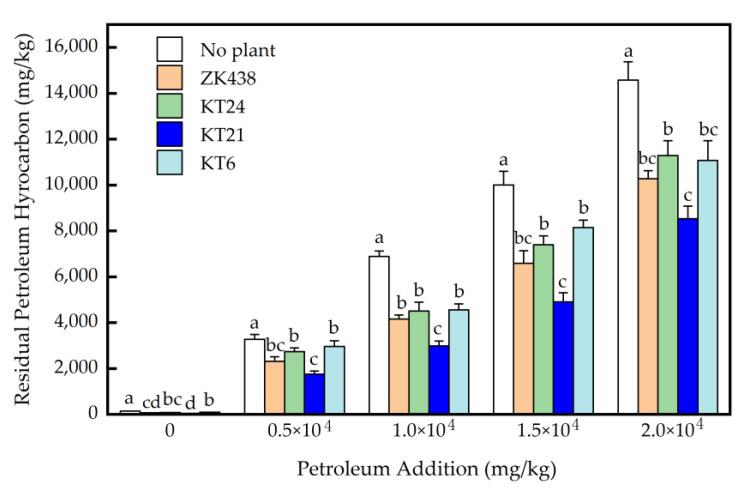
Residual petroleum hydrocarbons (C_10_-C_40_) in salinized soils after 70 days through the planting of the four *S. bicolor* varieties. (Different letters within the same petroleum addition indicated significant variances (*p* < 0.05) among different treatments.)

Based on the 70-day pot experiments, the removal of petroleum hydrocarbons in the salinized soils through the remediation of the four varieties, ZK438, KT24, KT21 and KT6, were studied (Figure 3). It was observed that the planting of the four varieties significantly reduced the contents of petroleum hydrocarbons in salinized soils compared with the control groups (*p* < 0.05). In salinized soils with petroleum hydrocarbon additions of 0, 5.0 × 10^3^, 1.0 × 10^4^ and 1.5 × 10^4^ mg/kg, the residuals of petroleum hydrocarbon in the soils where KT21 was growing were significantly lower than those with KT24 and KT6. In the salinized soils with 1.0 × 10^4^ mg/kg of petroleum added, the residuals of petroleum hydrocarbon in the soils with KT21 plants were significantly lower than those planted with ZK438. In the salinized soil with 0.5 × 10^4^, 1.0 × 10^4^, 1.5 × 10^4^ and 2.0 × 10^4^ mg/kg petroleum added, no significant difference in the residual levels of petroleum hydrocarbons was observed among the ZK438, KT24 and KT6 treatments (*p* > 0.05). Compared with the treatment without plants, the residual petroleum hydrocarbon in soils planted with KT21 decreased by 69.3%, 46.3%, 56.5%, 50.9% and 41.4%, for additions of 0, 0.5 × 10^4^, 1.0 × 10^4^, 1.5 × 10^4^ and 2.0 × 10^4^ mg/kg, respectively. Thus, KT21 was best for the remediation of petroleum in salinized oils.

## 4. Discussion

In the past three decades, phytoremediation has been widely investigated for the remediation of petroleum pollutants in soils due to its advantages of sustainability and ecological integrity [24,25,26,27]. The remediation of petroleum-contaminated soils by energy plants could not only solve the problem of land use, but may also prevent pollutants from entering the food chain, providing a new idea for the remediation of contaminated soil [9]. The effectiveness of phytoremediation technology depends on the synergistic relationship between plants, microorganisms and the soil environment. Different plant species or varieties of the same plant species have varied tolerance to petroleum hydrocarbons [28,29]. The seedling emergence rate and biomass are commonly used indicators of the toxic effects of contaminated soil on plants and are often used to screen plant varieties resistant to pollutants [30,31,32]. In this study, 23 of the 28 *S. bicolor* varieties tested were not affected by the addition of crude oils to salinized soil (*p* > 0.05). In salinized soil with petroleum additions of 0, 0.5 × 10^4^, 1.0 × 10^4^, 1.5 × 10^4^ and 2.0 × 10^4^ mg/kg, the emergence rate of KT24, KT6, ZK438 and KT21 was barely decreased (*p* > 0.05). Previous studies showed the emergence rate of gramineous plants seemed lower than that of other families in oil-contaminated soils [29], and oil contents >0.5 × 10^4^ mg/kg significantly reduced this parameter [33]. In this study, the emergence rate of sweet sorghum tended to be less affected by petroleum in salinized soil, which may be ascribed to sweet sorghum’s relatively large seeds. Large seeds usually have thicker seed coats and have a stronger resistance to petroleum hydrocarbon in soil [34]. In addition, large seeds can use their own energy stores to grow in soil contaminated by petroleum hydrocarbons, overcoming unfavorable conditions such as low fertility [35]. 

High stem and great below-ground biomass are considered the perfect characteristics of a phytoremediation plant species [33,36]. Sweet sorghum, a tall gramineous plant, has a larger root-specific surface area and high straw biomass [12]. These characteristics just meet the requirements for plant tools to repair contaminated soil. Among the 28 varieties tested, ZK438, KT24, KT21 and KT6 had a higher plant height and biomass in petroleum-contaminated salinized soil. Accompanying with strong tolerance to petroleum pollution and saline conditions, the capacity of degrading petroleum pollutants, especially toxic ones such as petroleum hydrocarbons, is the key to developing the phytoremediation potential of a plant variety [37,38,39]. A large number of studies show that planting plants can significantly improve the removal of petroleum hydrocarbon pollutants compared with non-planting controls [33,40,41,42,43]. For example, Li et al. reported the remediation effects of five species (*Gossypium hirsutum*, *Arachis hydropogaea*, *Capsella bursa-pastoris*, *Zinnia elegans* and *Allium ascalionicum*) on petroleum hydrocarbons, observing *A. hydropogaea* was the best plant species, removing >31% of the petroleum hydrocarbons in soil after a 70-day cultivation [44]. Cheng et al. investigated the remediation abilities of *Iris dicomoma* and *Iris lactea* to petroleum-contaminated saline–alkali soils and found *I. lactea* was more tolerant to petroleum pollution and could remove >20% of the pollutants [45]. Cai et al. reported *Testuca arundinacea* could remove >60% of the petroleum hydrocarbons in saline soil contained by crude oil after 90-day growth [46]. However, some research results show that compared with the controls without planting, planting plants failed to promote the effective degradation of petroleum hydrocarbon pollutants in soil [47,48]. Thus, the removal rate of petroleum hydrocarbons can vary among different species, and, of course, vary among different varieties. Our results showed that planting ZK438, KT24, KT21 and KT6 could effectively promote the removal of petroleum hydrocarbon in salinized soil. The removal rates of petroleum hydrocarbons in salinized soil after planting KT21 for 70 days were 41.4%–69.3% higher than those of the control groups, suggesting its great application potential in the remediation of oil-contaminated salinized soil.

In general, the four mechanisms of plant degradation, plant absorption, plant volatilization and rhizosphere degradation work during the phytoremediation process. The efficiency of phytoremediation mainly depends on the ability of plants to directly absorb and metabolize petroleum hydrocarbons and the ability to promote rhizosphere microbial remediation of petroleum hydrocarbons by releasing various secretions (sugars, amino acids, organic acids, etc.) and enzymes, and these factors largely depend on the genetic composition of plant species or varieties [49]. As for the phytoremediation of petroleum-contaminated soils, rhizosphere degradation was considered as the most important pathway for the removal of these pollutants since the bioavailability of petroleum hydrocarbons is very low and the biomass of harvested plants are relatively small, leading to a limited role for the above-ground portion of the plant [50]. In the present study, ZK438, KT24, KT21 and KT6 promoted the removal of petroleum pollutants in salinized soil to varying degrees, which might be related to the physiological and biochemical characteristics of different varieties of sweet sorghum. However, it is necessary to explore the removal mechanisms of hydrocarbons in the salinized soil planting varieties of *S. bicolor* in future studies. To sum up, the results of the present study provided preliminary information for using sweet sorghum to repair petroleum-contaminated salinized soil. Further field investigation should be conducted to prove these findings. 

## 5. Conclusions

Four varieties of sweet sorghum (ZK438, KT24, KT21 and KT6) resistant to petroleum pollution in salinized soil were selected from 28 commercial/research varieties in this study. The emergence rate of ZK438, KT24, KT21 and KT6 in salinized soil was not affected by the external petroleum with contents of 2.0 × 10^4^ mg/kg. Considering the fresh and dry weights of the above-ground and underground parts under different petroleum amounts in salinized soil, ZK438 and KT21 are more tolerant to oil pollutants than KT24 and KT6. Planting ZK438, KT24, KT21 and KT6 could effectively promote the removal of petroleum pollutants in salinized soil; of these, KT21 has the best remediation effects and show great application potential in the remediation of petroleum-contaminated salinized soil. 

## Figures and Tables

**Table 1:** Number of sweet sorghum varieties.

Variety	Number	Variety	Number	Variety	Number	Variety	Number
Jiliang No. 3	JL3	Liaotian No. 6	LT6	Ketian No. 11	KT11	JT No. 98	JT98
Zhong Ketian No. 438	ZK438	Gao Dancao	GD	Ketian No. 24	KT24	Ketian No. 17	KT17
Zhong Ketian No. 968	ZK968	Cao Gaoliang	CG	J No. 25	J25	2031	WZ20
Jinnuo No. 3	JN3	JT No. 47	JT47	Ketian No. 30	KT30	J100	J100
Jitian No. 3	JT3	JT No. 62	JT62	JT No. 110	JT110	WSC29	WS29
Longza No. 11	LZ11	Ketian No. 2	KT2	Ketian No. 21	KT21	Ketian No. 6	KT6
Ji Tianza No. 2	JT2	BJ No. 285	BJ285	Ketian No. 9	KT9	Ketian No. 4	KT4

**Table 2:** Primary physico–chemical properties of the tested soils.

pH (H_2_O)	Salinity (%)	Organic C (g/kg)	Total N (g/kg)	Total P (g/kg)	Available N (mg/kg)	Available P (mg/kg)	Petroleum Hydrocarbon (mg/kg)
7.85	0.31	10.78	0.79	0.87	35.53	29.09	146.67

## Data Availability

Not applicable.
